# Impact of Early Treatment with Pimavanserin on Healthcare Resource Utilization Among Newly Diagnosed Patients with Parkinson’s Disease Psychosis: A Pre-Post Medicare Claims Database Analysis

**DOI:** 10.36469/001c.154805

**Published:** 2026-01-23

**Authors:** Nazia Rashid, Krithika Rajagopalan, Daksha Gopal, Lambros Chrones, Dilesh Doshi

**Affiliations:** 1 Medical Affairs Acadia Pharmaceuticals (United States), San Diego, California; 2 Anlitiks Inc., Windermere, Florida; 3 Anlitiks Inc

**Keywords:** pimavanserin, Parkinson’s disease psychosis, healthcare resource utilization, Medicare claims, early treatment

## Abstract

**Background:**

Pimavanserin is currently the only atypical antipsychotic (AAP) approved by the US Food and Drug Administration for treating hallucinations and delusions in Parkinson’s disease psychosis (PDP). Benefit and efficacy of pimavanserin have been demonstrated through clinical trials; however, understanding other real-world outcomes is needed.

**Objective:**

This study evaluated healthcare resource utilization (HCRU) patterns before and after pimavanserin initiation to assess benefit and effectiveness of pimavanserin that may be seen in the real-world setting but not in clinical trials.

**Methods:**

A retrospective pre-post analysis using 100% Medicare claims data (Parts A, B, and D), April 1, 2015–December 31, 2021, was conducted. The study included AAP treatment–naïve PDP patients who initiated continuous pimavanserin monotherapy (index date) within 6 months of their incident PDP diagnosis, April 1, 2016–December 31, 2020 (ie, patient identification period). Outcomes measured during the 6 months before and after pimavanserin initiation included all-cause and psychiatric-related HCRU; further categorized as inpatient, emergency room (ER), outpatient, and office visits. Significant differences in the percentage of patients with at least 1 all-cause and at least 1 psychiatric-related HCRU were evaluated using McNemar’s tests at *P* < .05.

**Results:**

Of the 694 patients newly diagnosed with PDP who initiated pimavanserin within 6 months of PDP diagnosis, mean age was 76.9 (±6.8) years, 54.8% were male, and 78.3% had concomitant dementia. The percentage of patients with at least 1 all-cause HCRU was significantly higher during the 6 months pre-index vs post-index, with inpatient (26.1% vs 20.5%, *P* < .05), ER (51.3% vs 35.2%, *P* < .05), outpatient (83.0% vs 79.3%, *P* < .05), and office visits (97.4% vs 95.8%, *P* < .05). Similarly, the percentage of patients with at least 1 psychiatric-related HCRU was significantly higher 6 months pre-index vs post-index, with inpatient (7.8% vs 4.9%, *P* < .05), ER (9.7% vs 3.5%, *P* < .05), outpatient (23.6% vs 13.0%, *P* < .05), and office visits (68.7% vs 55.8%, *P* < .05).

**Conclusions:**

Newly diagnosed PDP patients initiating pimavanserin within 6 months demonstrated significant reductions in 6-month post-index all-cause and psychiatric-related HCRU. Further analysis examining the association between time of pimavanserin initiation and the magnitude of benefits are warranted.

## INTRODUCTION

Parkinson’s disease (PD) is a chronic, progressive neurodegenerative disorder that currently affects nearly 1 million individuals in the United States; this figure is projected to rise to approximately 1.6 million by 2037.^1,2^ Over the course of PD, approximately 50% of individuals are estimated to develop Parkinson’s disease psychosis (PDP), a debilitating condition characterized by hallucinations and delusions.^3^ In addition to PDP, cognitive impairment is frequently observed in patients with PD, with approximately 1 in 4 individuals exhibiting mild cognitive impairment at diagnosis and nearly 80% of them progressing to dementia within 20 years.^4^

Among prior published studies, PDP has been associated with significantly greater healthcare resource utilization (HCRU), including higher rates of inpatient hospitalizations, emergency room (ER) visits, and accelerated placement in long-term care (LTC) facilities compared with PD patients without psychosis.^5,6^ Other studies have demonstrated the increased risks of hospitalization, custodial care, and mortality among patients with PDP vs patients with PD alone.^7–9^ In addition to higher rates of HCRU, PDP patients enrolled in Medicare incurred higher healthcare costs than PD patients without psychosis; the greatest cost differences were observed among PDP patients in LTC facilities ($31 178 vs $14 461) and skilled nursing facilities (SNF) ($6601 vs $2067), and those with inpatient hospitalizations ($10 125 vs $6024).^5^ The study also suggested that, on average, nearly 75% of PDP patients resided 179 days in LTC compared with 56% of PD patients without psychosis, who resided 83 days.^5^

In 2016, pimavanserin (PIM) was the first atypical antipsychotic (AAP) to be approved by the US Food and Drug Administration (FDA) for the treatment for hallucinations and delusions associated with PDP.^10^ To date, PIM remains the only FDA-approved treatment for psychosis associated with PDP.^10^ PIM is a selective serotonin inverse agonist/antagonist targeting 5-HT2A receptors. It offers a favorable tolerability profile that may avoid PD-associated motor worsening symptoms, which remain a common concern with other AAPs.^11^ Published clinical trial results have also shown PIM’s efficacy in reducing psychotic symptoms while preserving motor function.^12^ The evidence-based review from the International Parkinson & Movement Disorder Society suggests that PIM is considered “clinically useful” in the treatment of PDP and may be a preferable first choice after optimization of antiparkinsonian medications.^13^

Real-world evidence has begun to illustrate that the potential benefits of PIM go beyond traditional “clinical usefulness” by lowering HCRU, including fewer inpatient hospitalizations and ER visits, and through delayed or lower likelihood of LTC or nursing home placement.^14–19^ Despite the established clinical efficacy and HCRU benefits of PIM among patients with PDP, little is known about the impact of timing of PIM treatment initiation on real-world HCRU outcomes. Published studies have suggested that delayed initiation of AAPs after PDP diagnosis may prolong patient and caregiver burden and increase HCRU among patients with PDP.^16,20^ Therefore, it is possible that early treatment with PIM may result in significant HCRU reduction, potentially mediated through more effective mitigation of psychotic symptoms before the acceleration of symptom severity.^21^

To provide quantitative data and address significant gaps in the literature regarding the impact of timing of PIM initiation after PDP diagnosis, this study examined the impact of PIM initiation among newly diagnosed PDP patients (ie, within 6 months of PDP diagnosis) on HCRU patterns, including inpatient and outpatient hospitalizations, ER visits, and office visits during the 6-month period following PIM initiation.

## METHODS

### Study Design and Data Source

A retrospective pre-post claims database analysis was conducted using a 100% Centers for Medicare and Medicaid Services (CMS) fee-for-service (FFS) sample including Part A (hospital insurance), Part B (medical insurance), and Part D (pharmacy) claims of Medicare beneficiaries between April 1, 2015, and December 31, 2021 (study period). This analysis was conducted in compliance with the Health Insurance Portability and Accountability Act (HIPAA) under a CMS data user agreement established pursuant to the New England Institutional Review Board’s review and approval.

### Study Population and Cohort Identification

Newly diagnosed PDP patients with at least 1 diagnostic claim for PD (ICD-9-CM: 332.0x; ICD-10-CM: G20.x) in combination with a documented diagnosis of psychosis were identified during the study period. Psychosis was defined based on claims for the following psychotic disorders in any diagnostic position (delusional disorder, ICD-9-CM: 297.1x; ICD-10-CM: F22.x; psychosis, ICD-9-CM: 298.0x, 298.1x, 298.4x, 298.8x, 298.9x; ICD-10-CM: F28.x, F29.x, F23.x; psychotic disorder with hallucinations or delusions, ICD-9-CM: 293.81, 293.82; ICD-10-CM: F06.0x, F06.2x) or hallucinations (ICD-9-CM: 780.1x; ICD-10-CM: R44.0x–R44.3x); or visual disturbances with hallucinations (ICD-9-CM: 368.16; ICD-10-CM: H53.16) (**Supplementary Table S1**).

Of the PDP patients, those with psychosis preceding PD diagnosis were excluded and the earliest psychosis claim following a PD diagnosis was considered the PDP diagnosis date for the selected PDP population. To ensure the capture of incident PDP, individuals with any psychosis diagnosis occurring before the initial PD diagnosis were excluded. Among the remaining population, only PDP patients with or without dementia, who had no evidence of antipsychotic exposure during the 12-month pre-index period (index date was defined as the first AAP prescription fill date), were included. Eligible individuals who initiated AAP monotherapy between April 1, 2016 (the month in which PIM was approved by the US FDA as a treatment for PDP), and December 31, 2020 (patient identification period), with a minimum 12-month pre-index (lookback) washout period and at least 6-month post-index follow-up were selected for this analysis.

The pre-index period was designed as an extended washout period for prior AAP use and to facilitate the appropriate assessment of baseline comorbidities. Additionally, the eligible patient population excluded patients with secondary parkinsonism, delirium, other psychotic disorders, substance-induced psychosis, schizophrenia, paranoia, or personality disorders during the pre-index period. Furthermore, to ensure that psychosis etiology was attributable to PD rather than to alternative causes, patients with baseline HIV, substance abuse, alcohol abuse, and psychosis, as defined by Elixhauser comorbidities, were excluded.^17^

**Figure 1.**
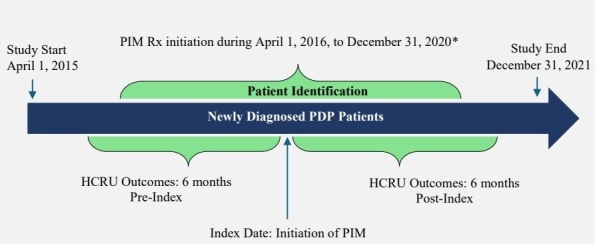
Study Schema Abbreviations: Dx, diagnosis; PDP, Parkinson’s disease psychosis; PIM, pimavanserin; Rx, prescription. *Patients were selected based on continuous enrollment throughout the study period, with HCRU outcomes evaluated over a 6-month period.

**Figure 2.**
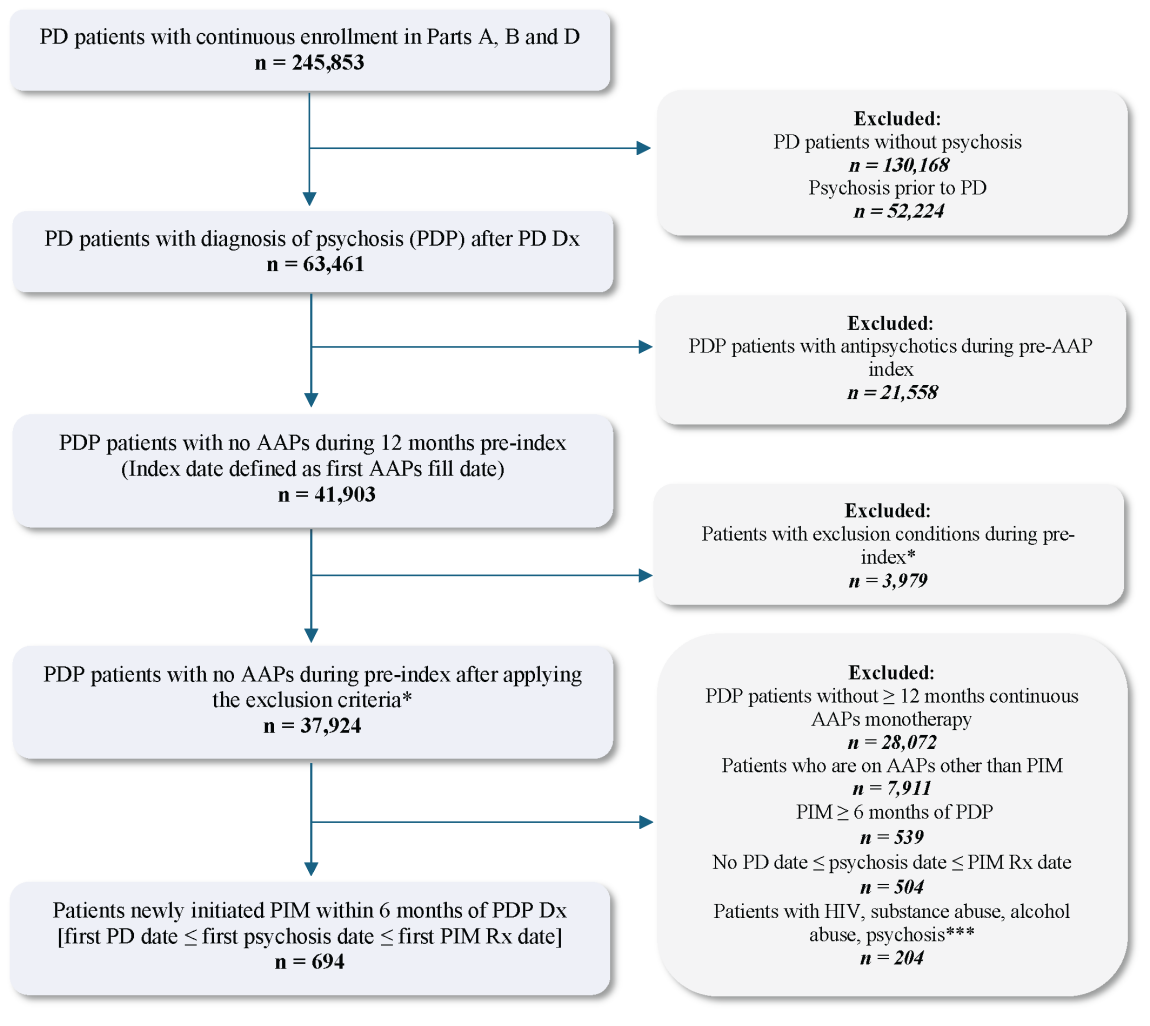
Patient Selection and Attrition Abbreviations: AAP, atypical antipsychotic; Dx, diagosis; PD, Parkinson’s disease; PDP, Parkinson’s disease psychosis; PIM, pimavanserin; Rx, prescription. *Diagnosis of secondary parkinsonianism, delirium, other psychotic disorder, alcohol-/drug-induced psychosis, schizophrenia, paranoia, or personality disorders. **Patients treated with AAPs included those treated with quetiapine, pimavanserin, risperidone, olanzapine, and aripiprazole; patients initiating clozapine, paliperidone, or brexipiprazole were not included due to small counts. ***Patients with baseline diagnosis codes for Elixhauser HIV, substance abuse, alcohol abuse, and psychosis were excluded.

From the eligible treatment-naïve and newly diagnosed PDP population selected above, patients who initiated PIM early (within 6 months of PDP diagnosis) and received monotherapy for at least 6 months were retained after excluding the following: (1) patients who initiated other AAP (ie, AAPs not approved for PDP) monotherapy for at least 6 months during the study identification period, and (2) patients who initiated PIM at least 6 months after PDP diagnosis. The PIM monotherapy period was defined by having continuous PIM prescription fills from Medicare Part D, covering the full 6-month post-index period, allowing for a 45-day grace period between refills. Any overlap with other AAPs during this 6-month period resulted in exclusion, ensuring PIM was the only AAP therapy. Lastly, to preserve correct temporal alignment of clinical events, PD diagnosis was required to occur on or before the PDP diagnosis, and PDP diagnosis was required to occur on or before PIM initiation. Patients were also required to have continuous (≥2 years) Medicare FFS enrollment and PIM monotherapy for the full 6-month pre-index and post-index periods. A detailed study design and final eligible sample along with the cohort selection criteria are shown in **Figure 1** and **Figure 2**, respectively. All ICD codes used for inclusion and exclusion criteria are provided in **Supplementary Table S1** and **Table S2**, respectively. The ICD coding algorithms used for the identification of PD and psychosis were adapted from previously published algorithms to ensure consistency with the established literature.^8,19^

### Study Covariates and HCRU Outcomes

Baseline demographic and clinical characteristics of the study cohort, including age, sex, race, geographic region, index year of PIM initiation, number of Elixhauser comorbidities, and other concomitant medications, were assessed during the 12-month pre-index period. ICD codes for comorbidities and drug class for concomitant medications are provided in **Supplementary Tables S3** and **Table S4**, respectively.

HCRU outcomes were evaluated during the 6-month period before and after PIM monotherapy initiation. Outcomes included all-cause and psychiatric-related HCRU. All-cause HCRU was defined as any HCRU admission or visit regardless of diagnosis claim position, whereas psychiatric-related HCRU was defined by claims with diagnosis for hallucinations, delusions, or psychosis in any diagnostic code position (**Supplementary Table S1**). Overall inpatient hospitalizations, along with type of hospitalizations (ie, short-term [ST], LTC, and SNF stays) as well as ER, outpatient, and office visits were identified based on claim type, place of service, and revenue center codes as outlined by the CMS and Chronic Conditions Data Warehouse guidelines.^22,23^ The CMS database reports inpatient hospitalizations overall as well as by hospitalization type, which is categorized by duration of stay. For example, ST stays were defined as hospital admissions of 25 days or less and were categorized as acute or critical care admissions. LTC stays were defined as admissions to LTC hospitals providing extended medical and rehabilitative care for patients with clinically complex conditions, with lengths of stay exceeding 25 days.^24^ SNF stays were identified as post-acute care stays of up to 100 days in SNFs, typically following a qualifying inpatient hospitalization and covered under Medicare Part A.^25^

Other HCRU outcomes included outpatient, ER, and office visits. Outpatient visits were defined as facility-based services covered by Medicare Part B, including those provided in outpatient departments in a hospital setting, ambulatory surgery centers, and observation stays of less than 24 hours.^22^ ER visits were defined as encounters at hospital emergency departments, which may or may not have resulted an inpatient admission.^23^ Office visits were defined as services delivered in physician offices or clinic settings. The same set of codes were used consistently across both the pre-index and post-index periods to ensure temporal consistency for all-cause and psychiatric-related HCRU outcomes.

### Statistical Methods

Descriptive statistics for patient characteristics and HCRU outcomes were reported as frequencies and percentages for categorical variables; mean, SD, median, and interquartile range (IQR) for continuous variables. Comparisons of HCRU outcomes between the pre-PIM and post-PIM periods were conducted using McNemar’s test, with statistical significance defined as *P* < .05. Patients were censored at the date of death or loss of Medicare FFS eligibility if either occurred during the 6-month post-index period. No imputation for missing data was performed. All analyses were performed using SAS Enterprise Server via the CMS Virtual Research Data Center.

## RESULTS

A total of 694 individuals who initiated PIM monotherapy within 6 months of a new PDP diagnosis formed the eligible sample for analysis. On average, the mean (SD) age of our study sample was 76.9 (6.8) years, and 54.8% were male. The majority of the eligible cohort was White (90.6%), followed by Black (2.7%), among other racial groups. Nearly half of the study cohort resided in the southern region (44.2%) of the United States, and the median number of Elixhauser comorbidities among the cohort was 4 (IQR, 2-6). The most common comorbidities included depression (33.7%), cardiac arrhythmia (21.2%), fluid and electrolyte disorders (17.9%), uncomplicated hypertension (66.7%), and other neurological disorders (98.9%). Other baseline comorbidities observed in our cohort included congestive heart failure (10.4%) and chronic obstructive pulmonary disease (11.1%). Approximately 1 in 3 patients had coexisting insomnia (37.3%), and 4 in 5 patients had coexisting dementia (78.3%). A complete list of patient demographics and baseline comorbidities is shown in **Table 1** and **Table 2**, respectively. During baseline, nearly one-third were receiving antidementia medications (32.7%), benzodiazepines (29.2%), and selective serotonin reuptake inhibitors (30.9%). On the other hand, monoamine oxidase inhibitors were used in 21.5% of patients, serotonin and norepinephrine reuptake inhibitors were used by 12.1% of patients, and 18.7% received other antidepressants. The list of patients on concomitant medications in the study cohort at baseline are shown in **Table 1**.

**Table 1.** Demographic Characteristics of Patients With Parkinson’s Disease Psychosis Initiating Pimavanserin Within 6 Months of Diagnosis

**Demographic Characteristic**	**PIM Initiators (N = 694)**
Age (y), mean (SD)	76.9 (6.8)
Gender, n (%)	
Male	380 (54.8)
Index year of PIM initiation, n (%)	
2016	82 (11.8)
2017	167 (24.0)
2018	132 (19.0)
2019	170 (24.5)
2020	143 (20.6)
Race, n (%)	
White	629 (90.6)
Black	19 (2.7)
Unknown	a
Other	15 (2.2)
Asian	13 (1.9)
Hispanic	a
American Native	a
Geographic region, n (%)	
Midwest	142 (20.5)
Northeast	125 (18.0)
South	307 (44.2)
West	120 (17.3)
No. of Elixhauser comorbidities	
Mean (SD)	4.2 (2.5)
Median (IQR)	4 (2-6)
Other concomitant comorbidities, n (%)	
Dementia	544 (78.3)
Insomnia	259 (37.3)
Baseline medications, n (%)	
Antidementia	227 (32.7)
Rivastigmine	89 (12.8)
Donepezil	151 (21.7)
Benzodiazepine	203 (29.2)
Anticonvulsant/mood stabilizer	21 (3.0)
SSRI	215 (30.9)
SNRI	84 (12.1)
TCA	14 (2.0)
MOAI	149 (21.5)
Other antidepressant	130 (18.7)

Abbreviations: IQR, interquartile range; MAOI, monoamine oxidase inhibitor; PIM, pimavanserin; SNRI, serotonin and norepinephrine reuptake inhibitor; SSRI, selective serotonin reuptake inhibitor; TCA, tricyclic antidepressant.

^a^Cell sizes <11 are suppressed as per CMS guidelines.

**Table 2.** Comorbidities Among Patients With Parkinson’s Disease Psychosis Initiating Pimavanserin Within 6 Months of Diagnosis

**Clinical Comorbidities, n (%)**	**PIM Initiators (N = 694)**
Blood loss anemia	b
Cardiac arrhythmia	147 (21.2)
Chronic pulmonary disease	77 (11.1)
Coagulopathy	26 (3.7)
Congestive heart failure	72 (10.4)
Deficiency anemia	65 (9.4)
Depression	234 (33.7)
Diabetes, complicated	95 (13.7)
Diabetes, uncomplicated	124 (17.9)
Fluid and electrolyte disorders	124 (17.9)
Hypertension, complicated	100 (14.4)
Hypertension, uncomplicated	463 (66.7)
Hypothyroidism	140 (20.2)
Liver disease	14 (2.0)
Lymphoma	b
Metastatic cancer	b
Obesity	44 (6.3)
Other neurological disorders^a^	686 (98.9)
Paralysis	b
Peptic ulcer disease excluding bleeding	b
Peripheral vascular disease	131 (18.9)
Pulmonary circulation disorder	20 (2.9)
Renal failure	92 (13.3)
Rheumatoid arthritis	30 (4.3)
Solid tumors without metastasis	50 (7.2)
Valvular disease	76 (11.0)
Weight loss	61 (8.8)

^a^Multiple sclerosis, other demyelinating diseases of central nervous system, epileptic seizures, other central nervous system disorders.

^b^Cell sizes <11 are suppressed as per CMS guidelines.

**Figure 3.**
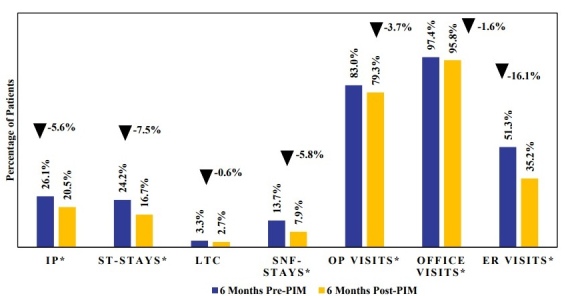
All-Cause HCRU Among Patients With Parkinson’s Disease Psychosis Initiating Pimavanserin Within 6 Months of Diagnosis Abbreviations: ER, emergency room; HCRU, healthcare resource utilization; IP, inpatient; LTC, long-term care, OP, outpatient; PIM, pimavanserin; SNF, skilled nursing facility; ST, short-term. *Statistically significant at *P* < .05.

All-cause inpatient hospitalization rates were lower in the 6-month post-index period following PIM initiation compared with the 6-month pre-index period, across all hospitalization types (**Figure 3; Supplementary Table S5**). The proportion of patients with at least 1 inpatient hospitalization decreased from 26.1% pre-PIM to 20.5% post-PIM (*P* = .0075). This reduction was primarily driven by a decrease in ST stays (24.2% vs 16.7%, *P* = .0002). LTC stays showed a nonsignificant reduction (3.3% vs 2.7%), while SNF stays decreased significantly from 13.7% to 7.9% (*P* = .0002). Outpatient visits (83.0% vs 79.3%, *P* = .0398) and office visits (97.4% vs 95.8%, *P* = .0343) also declined during post-PIM index period compared with the pre-PIM index period. The largest reduction was seen in ER visits, dropping from 51.3% pre-PIM to 35.2% post-PIM (*P* < .0001).

**Figure 4.**
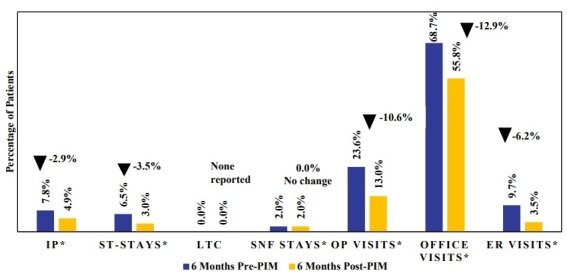
Psychiatric-Related HCRU Among Patients With Parkinson’s Disease Psychosis Initiating Pimavanserin Within 6 Months of Diagnosis Abbreviations: ER, emergency room; HCRU, healthcare resource utilization; IP, inpatient; LTC, long-term care, OP, outpatient; PIM, pimavanserin; SNF, skilled nursing facility; ST, short-term. *Statistically significant at *P *< .05.

Psychiatric-related inpatient hospitalization rates were also lower in the 6-month period following PIM initiation compared with the 6-month pre-index period across most hospitalization types (**Figure 4; Supplementary Table S5**). Psychiatric-related inpatient hospitalizations declined from 7.8% pre-PIM to 4.9% post-PIM (*P* = .0218). Additionally, psychiatric-related ST stays decreased from 6.5% pre-PIM to 3.0% post-PIM (*P* = .0016); however, psychiatric-related LTC stays were infrequent and did not show a significant difference. No change was observed in psychiatric-related SNF-stays (2.0% vs 2.0%) between the pre-and post-PIM periods. Psychiatric-related outpatient visits decreased from 23.6% to 13.0% (*P* < .0001), and psychiatric-related ER visits declined from 9.7% to 3.5% (*P* < .0001). Significant reductions were also seen in psychiatric-related office visits, from 68.7% in the pre-PIM period to 55.8% post-PIM (*P* < .0001).

## DISCUSSION

This pre-post analysis is the first of its kind to evaluate the immediate impact of PIM initiation among newly diagnosed PDP patients during 6-month follow-up. These findings show that initiating PIM within 6 months of PDP diagnosis was associated with significant reductions in all-cause and psychiatric-related HCRU during the post-PIM initiation period compared with the pre-PIM initiation period. Given the debilitating nature of hallucinations and delusions associated with PDP, the timely initiation of PIM may play a key role in reducing the HCRU burden of PDP, which is often associated with complex management needs and frequent healthcare encounters.^14–18^ Since PIM was initiated among newly diagnosed PDP patients in this analysis, these results may support PIM’s potential role as a first-line treatment to mitigate the psychotic symptoms as recommended in the IPMDS guidelines.^13,16^

Reductions were observed in all HCRU outcome measures (inpatient, ER, outpatient, office visits) in the post-PIM initiation period. However, the largest reductions were observed in all-cause ER visits and inpatient hospitalizations. Specifically, ER visits decreased by 16.1% and inpatient hospitalizations decreased by 5.6%, with the latter primarily driven by declines in ST stays (7.5%) and SNF stays (5.8%). It is possible that early treatment with PIM and its immediate impact on hallucinations and delusions may result in fewer ST stays, as seen in this analysis. It is also plausible that the maintenance of treatment effect with PIM over the long term may be a driver of reduced SNF stays of up to 100 days. These results are consistent with prior studies showing that patients treated with PIM experience fewer ER visits and hospitalizations, including fewer ST stays and SNF stays compared with those receiving other AAPs.^14–18^ Given that PDP patients have been shown to incur 2.3 times greater HCRU rates compared with patients with PD only,^6^ the observed HCRU reductions with PIM initiation may carry both clinical and economic significance. While claims data inherently lack clinical information related to patient symptomatology, it is plausible that the reduced need for ER visits and inpatient hospitalizations with the use of PIM shortly after PDP diagnosis may be mediated through PIM’s response effect in the reduction of psychotic symptoms. The pattern of decreased utilization across multiple settings suggests that early initiation of PIM may stabilize symptoms of hallucinations and delusions, thereby reducing the need for acute care encounters. These results are particularly relevant given the high rates of hospitalization and LTC placement among PDP patients.^8^ Supporting our hypotheses, a recent post hoc analysis of randomized trial data presented at Psych Congress 2024 further reinforces this observation, where patients who initiated PIM within 6 months of PDP onset were more than 3 times likely to achieve complete symptom resolution compared with later initiators, with benefits evident within the first month and sustained for a year.^26^ Together, these findings may highlight the potential benefits of early intervention with PIM.^26^

Despite the progressive nature of PDP, nearly half the patients in our study initiated PIM at least 6 months after their PDP diagnosis. While it is unclear why PIM initiation was delayed among these patients, it is possible that clinicians may delay treatment when PDP symptoms are perceived to be less severe.^27^ Alternatively, it is also possible that physicians who delay PIM initiation may be non-PD specialists or community practitioners, have less clinical experience in treating PDP, or lack awareness about PIM’s clinical benefits and its approved indication for PDP. This hypothesis, however, requires future evaluation to understand the physician profile of PIM prescribers who delay treatment initiation. These results also suggest the increased need for continuing provider medical education about the role of PIM as a first-line treatment option in PDP as per the International Parkinson & Movement Disorder Society guideline recommendations.

Based on clinician input in our analysis, a follow-up treatment interval of 6 months was considered adequate to examine the impact of PIM initiation among PDP patients because it was anticipated that at least 6 weeks of treatment with PIM is necessary to demonstrate its full therapeutic effect.^21^ Furthermore, it was expected that the 6-month window would minimize the potential for time-varying confounding and treatment switching that may be more prevalent during a longer, 12-month follow-up period.

Future studies may be needed to examine if different initiation time (ie, alternative definitions of early treatment) and follow-up periods may yield different results or if modeling treatment initiation as a continuous variable may help us better capture the gradual progression of symptom severity and its correlation with PIM’s impact on HCRU. Overall, such research would provide greater insights into whether the magnitude of HCRU benefit varies by timing of PIM initiation, the length of follow-up, and the type of population subgroups. Additionally, the role of PIM as a first-line therapy may be further explored to assess its broader impact on disease trajectory, symptom management, and longer-term HCRU outcomes. Lastly, the prescriber profile of PIM warrants further investigation into understanding the role of clinical experience and association with the timing of PIM prescribing among patients with PDP patients.

### Limitations

As with all retrospective analyses using administrative claims data, the limitations associated with this study should be considered when interpreting the current findings. This study has limitations inherent to a single group pre-post observational design. Given the absence of a comparator group in this analysis, the observed changes in HCRU may not be attributable to PIM treatment only. It is possible that differences in HCRU patterns between the pre- and post-initiation periods may also reflect potential progression of the disease or increased clinical attention following the diagnosis of PDP rather than a direct treatment effect. However, it is believed that the HCRU reduction occurring for any cause as well as PDP-related causes is indicative of a true treatment effect of PIM. The authors, however, acknowledge that regression to the mean and the role of time-varying confounders (eg, changes in dopaminergic medication dose, concomitant medications) that are not observable in claims data such as the Medicare sample may not be ruled out. It should also be noted that pre-post designs generally mitigate this limitation by utilizing each patient as their own control, thereby providing insights about the HCRU trends before and after PIM treatment.

By limiting the follow-up period to 6 months instead of a longer period, however, we aimed to limit the potential for time-varying confounders. It should also be noted that the pre-period utilization in this analysis could reflect background healthcare use rather than PDP burden since part of the 6-month pre-index period could occur before PDP was clinically recognized in some patients. Additionally, it is possible that survivorship bias, a form of selection bias, resulting from the study requirement to include only patients with at least 6 months of PIM monotherapy (ie, patients who survive and remain on therapy long enough) may limit our findings. Given these limitations, the results should be interpreted as descriptive associations rather than causal estimates. Furthermore, results of this analysis may also be biased due to diagnostic coding errors related to undercoding or miscoding and potential residual confounding due to unobservable clinical characteristics such as disease severity, functional status, that may influence outcomes. Finally, the results from the analysis of Medicare patients may not be applicable to younger patients or those with different insurance coverage.

Despite these limitations, the findings provide valuable real-world evidence supporting the potential benefits of PIM initiation and its immediate impact among newly diagnosed PDP patients.

## CONCLUSION

In this retrospective real-world claims database analysis of Medicare patients with PDP, initiation of PIM within 6 months of PDP diagnosis was associated with significant reductions in the percentage of patients with all-cause and psychiatric-related HCRU in the 6-month post-index period compared with the 6-month pre-index period. These findings suggest that early treatment with PIM may reduce the HCRU burden in the PDP population. Further research may be needed to explore the association between timing of PIM initiation after psychosis onset and the extent of clinical benefits as well as the role of prescriber clinical experience in the timing of PIM initiation among PDP patients. These potential future investigations may be helpful in expanding the understanding of the role of PIM as a first-line treatment for patients with PDP.

### Disclosures

N.R., L.C., and D.D. are employees of Acadia Pharmaceuticals. D.K. and K.R. are employees of Anlitiks Inc., which received funding from Acadia Pharmaceuticals to conduct this study.

## Supplementary Material

Online Supplementary Material
