# Supplementary material for: Impact of Early Treatment with Pimavanserin on Healthcare Resource Utilization Among Newly Diagnosed Patients with Parkinson’s Disease Psychosis: A Pre-Post Medicare Claims Database Analysis
Source: J Health Econ Outcomes Res. 2026 Jan 23;13(1):1–9. doi: 10.36469/001c.154805 (PMC12832090; doi:10.36469/001c.154805)
Supplement: Online Supplementary Material [file jheor_2026_13_1_154805_324195.pdf]

Journal of Health Economics and Outcomes Research

Online Supplementary Material

Impact of Early Treatment with Pimavanserin on Healthcare Resource Utilization Among Newly Diagnosed Patients with Parkinson's Disease Psychosis: A Pre-Post Medicare Claims Database Analysis. *JHEOR*. 2026;13(1):1-9. doi:10.36469/jheor.2026.154805

Table S1: ICD Codes Used in the Study for Inclusion and Additional Comorbidities

Table S2. ICD Codes Used in the Study for Exclusion

Table S3. ICD-9 and ICD-10 Codes to Calculate Elixhauser Comorbidity

Table S4. Concomitant Medications List Used at Baseline

Table S5. All-Cause and Psychiatric-Related HCRU Among Patients Who Initiated Pimavanserin Within 6 Months of PDP (n = 694)

This supplementary material has been provided by the authors to give readers additional information about their work.

This is an open-access article distributed under the terms of the Creative Commons Attribution 4.0 International License (CCBY-4.0). View this license's legal deed at http://creativecommons.org/licenses/by/4.0 and legal code at http://creativecommons.org/licenses/by/4.0/legalcode for more information.

Table S1. ICD Codes Used in the Study for Inclusion and Additional Comorbidities

**Description**	**ICD-9-CM**	**ICD-10-CM**
Psychosis		
Delusion disorder	297.1x	F22.x
Hallucinations	780.1x	R44.0x, R44.1x, R44.2x, R44.3x
Psychosis	298.9x, 298.0x, 298.1x, 298.4x, 298.8x	F28.x, F29.x, F23.x
Psychotic disorder with hallucination/delusions	293.81, 293.82	F06.0x, F06.2x
Visual disturbances	368.16	H53.16
Additional comorbidities		
Dementia	331.x, 290.4x, 290.40, 290.41, 290.42, 331.82, 331.1x, 331.11, 311.19, 290.x, 290.0x, 290.1x, 290.1x, 290.10, 290.11, 290.12, 290.13, 290.20, 290.21, 290.3x, 290.8x, 290.9x, 294.1x, 294.10, 294.11, 294.2x, 294.20, 294.21, 331.6x	G30.x, G30.0x, G30.1x, G30.8x, G30.9x, F01.x, F01.5x, F01.50, F01.51, G31.83, G31.0x, G31.01, G31.09, G31.1x, G31.85, F03.x, F03.9x, F03.90, F03.91, F02.x, F02.8, F02.80, F02.81
Insomnia	307.41, 307.42, 327.x, 327.01, 327.02, 327.09, 780.51, 780.52	F51.01, F51.02, F51.03, F51.04, F51.05, F51.09, G47.00, G47.01, G47.09

Abbreviation: ICD, *International Classification of Diseases, Clinical Modification.*

Table S2. ICD Codes Used in the Study for Exclusion

**Description**	**ICD-9-CM**	**ICD-10-CM**
Alcohol-induced psychosis	291.0x, 291.3x, 291.5x	F10.121, F10.150, F10.151, F10.159, F10.221, F10.231, F10.250, F10.251, F10.259, F10.921, F10.950, F10.951, F10.959
Delirium	292.81, 293.0x, 293.1x, 293.83	
Drug-induced psychosis	292.11, 292.12, 292.81	F11.121, F11.150, F11.151, F11.159, F11.221, F11.250, F11.251, F11.259, F11.921, F11.950, F11.951, F11.959, F12.121, F12.150, F12.151, F12.159, F12.221, F12.250, F12.251, F12.259, F12.921, F12.950, F12.951, F12.959, F13.121, F13.150, F13.151, F13.159, F13.221, F13.231, F13.250, F13.251, F13.259, F13.921, F13.931, F13.950, F13.951, F13.959, F14.121, F14.150, F14.151, F14.159, F14.221, F14.250, F14.251, F14.259, F14.921, F14.950, F14.951, F14.959, F15.121, F15.150, F15.151, F15.159, F15.221, F15.250, F15.251, F15.259, F15.921, F15.950, F15.951, F15.959, F16.121, F16.122, F16.150, F16.151, F16.159, F16.183, F16.221, F16.250, F16.251, F16.259, F16.283, F16.921, F16.950, F16.951, F16.959, F16.983, F18.121, F18.150, F18.151, F18.159, F18.221, F18.250, F18.250, F18.251, F18.259, F18.921, F18.950, F18.951, F18.959, F19.121, F19.122, F19.150, F19.151, F19.159, F19.221, F19.231, F19.250, F19.251, F19.259, F19.921, F19.931, F19.939, F19.94, F19.950, F19.951, F19.959
Other psychotic conditions	290.8x, 290.9x	F32.3x, F33.3x
Paranoia	297.0x, 297.3x, 297.8x, 297.9x, 298.3x	

Table S2. ICD Codes Used in the Study for Exclusion

Description	ICD-9-CM	ICD-10-CM
Secondary parkinsonism	332.1x	G21.x, G21.0x, G21.1x, G21.11, G21.19, G21.18, G21.2x, G21.3x, G21.4x, G21.9x
Schizophrenia	295.00, 295.01, 295.02, 295.03, 295.04, 295.05, 295.10, 295.11, 295.12, 295.13, 295.14, 295.15, 295.20, 295.21, 295.22, 295.23, 295.24, 295.25, 295.30, 295.31, 295.32, 295.33, 295.34, 295.35, 295.40, 295.41, 295.42, 295.43, 295.44, 295.45, 295.50, 295.51, 295.52, 295.53, 295.54, 295.55, 295.60, 295.61, 295.62, 295.63, 295.64, 295.65, 295.70, 295.71, 295.72, 295.73, 295.74, 295.75, 295.80, 295.81, 295.82, 295.83, 295.84, 295.85, 295.90, 295.91, 295.92, 295.93, 295.94, 295.95	F20.x, F20.0x, F20.1x, F20.2x, F20.3x, F20.5x, F20.8x, F20.81, F20.89, F20.9x, F21.x, F24.x, F25.0x, F25.1x, F25.8x, F25.9x, V11.0x

Abbreviation: ICD, *International Classification of Diseases, Clinical Modification*.

Table S3. ICD-9 and ICD-10 Codes to Calculate Elixhauser Comorbidity

Comorbidity	ICD-9 Codes	ICD-10 Codes
AIDS/HIV	042.x - 044.x	B20.x - B22.x, B24.x
Blood loss anemia	280.0	D50.0
Coagulopathy	286.x, 287.1, 287.3 - 287.5	D65 - D68.x, D69.1, D69.3 - D69.6
Congestive heart failure	398.91, 402.01, 402.11, 402.91, 404.01, 404.03, 404.11, 404.13, 404.91, 404.93, 425.4 - 425.9, 428.x	I09.9, I11.0, I13.0, I13.2, I25.5, I42.0, I42.5 - I42.9, I43.x, I50.x, P29.0
Cardiac arrhythmias	426.0, 426.13, 426.7, 426.9, 426.10, 426.12, 427.0 - 427.4, 427.6 - 427.9, 785.0, 996.01, 996.04, V45.0, V53.3	I44.1 - I44.3, I45.6, I45.9, I47.x - I49.x, R00.0, R00.1, R00.8, T82.1, Z45.0, Z95.0
Chronic pulmonary disease	416.8, 416.9, 490.x - 505.x, 506.4, 508.1, 508.8	I27.8, I27.9, J40.x - J47.x, J60.x - J67.x, J68.4, J70.1, J70.3
Diabetes, uncomplicated	250.0 - 250.3	E10.0, E10.1, E10.9, E11.0, E11.1, E11.9, E12.0, E12.1, E12.9, E13.0, E13.1, E13.9, E14.0, E14.1, E14.9
Diabetes, complicated	250.4 - 250.9	E10.2 - E10.8, E11.2 - E11.8, E12.2 - E12.8, E13.2 - E13.8, E14.2 - E14.8
Deficiency anemia	280.1 - 280.9, 281.x	D50.8, D50.9, D51.x - D53.x
Depression	296.2, 296.3, 296.5, 300.4, 309.x, 311	F20.4, F31.3 - F31.5, F32.x, F33.x, F34.1, F41.2, F43.2
Fluid and electrolyte disorders	253.6, 276.x	E22.2, E86.x, E87.x
Hypertension, uncomplicated	401.x	I10.x
Hypertension, complicated	402.x - 405.x	I11.x - I13.x, I15.x
Hypothyroidism	240.9, 243.x, 244.x, 246.1, 246.8	E00.x - E03.x, E89.0
Liver disease	070.22, 070.23, 070.32, 070.33, 070.44, 070.54, 070.6, 070.9, 456.0 - 456.2, 570.x, 571.x, 572.2 - 572.8, 573.3, 573.4, 573.8, 573.9, V42.7	B18.x, I85.x, I86.4, I98.2, K70.x, K71.1, K71.3 - K71.5, K71.7, K72.x - K74.x, K76.0, K76.2 - K76.9, Z94.4
Lymphoma	200.x - 202.x, 203.0, 238.6	C81.x - C85.x, C88.x, C96.x, C90.0, C90.2
Metastatic cancer	196.x - 199.x	C77.x - C80.x
Obesity	278.0	E66.x

Table S3. ICD-9 and ICD-10 Codes to Calculate Elixhauser Comorbidity

Comorbidity	ICD-9 Codes	ICD-10 Codes
Other neurological disorders	331.9, 332.0, 332.1, 333.4, 333.5, 333.92, 334.x - 335.x, 336.2, 340.x, 341.x, 345.x, 348.1, 348.3, 780.3, 784.3	G10.x - G13.x, G20.x - G22.x, G25.4, G25.5, G31.2, G31.8, G31.9, G32.x, G35.x - G37.x, G40.x, G41.x, G93.1, G93.4, R47.0, R56.x
Paralysis	334.1, 342.x, 343.x, 344.0 - 344.6, 344.9	G04.1, G11.4, G80.1, G80.2, G81.x, G82.x, G83.0 - G83.4, G83.9
Peripheral vascular disorders	093.0, 437.3, 440.x, 441.x, 443.1 - 443.9, 447.1, 557.1, 557.9, V43.4	I70.x, I71.x, I73.1, I73.8, I73.9, I77.1, I79.0, I79.2, K55.1, K55.8, K55.9, Z95.8, Z95.9
Peptic ulcer disease, excluding bleeding	531.7, 531.9, 532.7, 532.9, 533.7, 533.9, 534.7, 534.9	K25.7, K25.9, K26.7, K26.9, K27.7, K27.9, K28.7, K28.9
Pulmonary circulation disorders	415.0, 415.1, 416.x, 417.0, 417.8, 417.9	I26.x, I27.x, I28.0, I28.8, I28.9
Renal failure	403.01, 403.11, 403.91, 404.02, 404.03, 404.12, 404.13, 404.92, 404.93, 585.x, 586.x, 588.0, V42.0, V45.1, V56.x	I12.0, I13.1, N18.x, N19.x, N25.0, Z49.0 - Z49.2, Z94.0, Z99.2
Rheumatoid arthritis/collagen vascular diseases	446.x, 701.0, 710.0 - 710.4, 710.8, 710.9, 711.2, 714.x, 719.3, 720.x, 725.x, 728.5, 728.89, 729.30	L94.0, L94.1, L94.3, M05.x, M06.x, M08.x, M12.0, M12.3, M30.x, M31.0 - M31.3, M32.x - M35.x, M45.x, M46.1, M46.8, M46.9
Solid tumor without metastasis	140.x - 172.x, 174.x - 195.x	C00.x - C26.x, C30.x - C34.x, C37.x - C41.x, C43.x, C45.x - C58.x, C60.x - C76.x, C97.x
Weight loss	260.x - 263.x, 783.2, 799.4	E40.x - E46.x, R63.4, R64
Valvular disease	093.2, 394.x - 397.x, 424.x, 746.3 - 746.6, V42.2, V43.3	A52.0, I05.x - I08.x, I09.1, I09.8, I34.x - I39.x, Q23.0 - Q23.3, Z95.2 - Z95.4

Abbreviation: ICD, *International Classification of Diseases, Clinical Modification*.

Table S4. Concomitant Medications List Used at Baseline

Drug Class	Drug List
Anticonvulsant/mood stabilizers	Carbamazepine, divalproex, divalproex sodium, lamotrigine
Antidementia (acetylcholinesterase inhibitors)	Rivastigmine, rivastigmine tartrate, donepezil HCl, donepezil HCl, tacrine HCl, tacrine HCl, galantamine hydrobromide
Benzodiazepines	Alprazolam, alprazolam, extended-release, chlordiazepoxide HCl, chlordiazepoxide HCl, clonazepam, clorazepate dipotassium, diazepam, lorazepam, oxazepam
Monoamine oxidase inhibitors	Selegiline, selegiline HCl, rasagiline, rasagiline mesylate, safinamide, safinamide mesylate
Other antidepressants	Bupropion hydrobromide, bupropion HCl, maprotiline HCl, maprotiline HCl, mirtazapine, nefazodone, trazodone HCl, trazodone HCl, vilazodone HCl, vilazodone HCl, vortioxetine, vortioxetine hydrobromide, doxepin HCl oral, doxepin HCl, doxepin HCl
Selective serotonin reuptake inhibitors	Escitalopram oxalate, fluoxetine HCl, fluoxetine HCl, fluvoxamine maleate, hypericum perforatum, paroxetine HCl, paroxetine HCl, paroxetine mesylate, sertraline HCl, sertraline HCl
Serotonin and norepinephrine reuptake inhibitors	Desvenlafaxine, desvenlafaxine succinate, duloxetine HCl, duloxetine HCl, levomilnacipran, venlafaxine HCl, venlafaxine HCl
Tricyclic antidepressants	Amitriptyline HCl, amitriptyline HCl, amitriptyline HCl, amoxapine, clomipramine HCl, clomipramine HCl, desipramine HCl, desipramine HCl, imipramine HCl, imipramine HCl, imipramine pamoate, nortriptyline HCl, nortriptyline HCl, protriptyline HCl, protr protriptyline iptyline HCl, trimipramine maleate

Table S5. All-Cause and Psychiatric-Related HCRU Among Patients Who Initiated PIM Within 6 Months of PDP (n = 694)

HCRU Setting	All-Cause (≥1 HCRU) Event						Psychiatric-Related (≥1 HCRU) Event					
	Pre-PIM, 6 Months		Post-PIM, 6 Months		% Change	*P*	Pre-PIM, 6 Months		Post-PIM, 6 Months		% Change	*P*
	N	%	N	%			N	%	N	%		
IP hospitalizations	181	26.1	142	20.5	-5.6	.0075	54	7.8	34	4.9	-2.9	.0218
Short-term stays	168	24.2	116	16.7	-7.5	.0002	45	6.5	21	3.0	-3.5	.0016
LTC stays	23	3.3	19	2.7	-0.6	NS	a	a	a	a	-	NS
SNF stays	95	13.7	55	7.9	-5.8	.0002	14	2.0	14	2.0	0	NS
Outpatient visits	576	83.0	550	79.3	-3.7	.0398	164	23.6	90	13.0	-1.6	<.0001
Office visits	676	97.4	665	95.8	-1.6	.0343	477	68.7	387	55.8	-12.9	<.0001
ER visits	356	51.3	244	35.2	-16.1	<.0001	67	9.7	24	3.5	-6.2	<.0001

Abbreviations: ER, emergency room; HCRU, healthcare resource utilization; IP, inpatient; LTC, long-term care; NS, not significant; PIM, pimavanserin; SNF, skilled nursing facility.
[a]Cell sizes <11 are suppressed as per Center for Medicare and Medicaid Services guidelines.
